# The utilization of renal dialysis: a comprehensive study in Saudi Arabia

**DOI:** 10.1186/s12889-024-19450-5

**Published:** 2024-07-16

**Authors:** Loujain S. Alghamdi, Wadi Alonazi

**Affiliations:** Health Administration Department, College of Business Administration, King Saud University, Riyadh, Riyadh, 13242-8424 Saudi Arabia

**Keywords:** Renal dialysis, Quality of care, Chronic renal failure, Saudi Arabia

## Abstract

**Background:**

Understanding the trend of utilization of renal dialysis in Saudi Arabia (SA) is fundamental as it provides a general overview of renal care. The practice of renal dialysis assists in identifying challenges, opportunities, and potential areas for improvement in the provision of the services.

**Objectives:**

This research investigated the utilization of renal dialysis services in SA by exploring the number of renal dialysis centers, hemodialysis machines (HD), and peritoneodialysis patients.

**Methods:**

The dataset for this study was derived from a collaboration between the General Authority of Statistics (GaStat) and the Ministry of Health (MoH), focusing on indicators for renal dialysis centers and patients across health sectors in 2021. Analysis was conducted using MS Excel 365 and IBM SPSS Version 29, incorporating multiple regression techniques. The health sector was treated as the dependent variable. At the same time, the number of hemodialysis (HD) machines and the counts of HD and peritoneal dialysis patients were considered independent variables.

**Results:**

Around 275 renal dialysis centers, over 8000 HD machines, 20,440 HD patients, and 1,861 peritoneal patients were tallied from two resources. The findings revealed a negative relationship between the health sector and several renal dialysis centers and peritoneodialysis patients, as demonstrated by *p* < 0.05 in multiple regression analysis.

**Conclusion:**

The number of renal dialysis centers influences the availability of HD machines, affecting the number of HD and peritoneodialysis patients. Most national patients preferred MoH over other semi-governmental and private sectors, and vice versa for non-Saudis.

## Introduction

The concept of renal dialysis, a medical procedure designed to eliminate waste products and surplus fluids from the bloodstream in cases of renal dysfunction, originated and was first implemented during the early 1940s [1]. Willem Kolff, a distinguished Dutch physician, is recognized for constructing the initial functional dialyzer in 1943, a period marked by the Nazi occupation of the Netherlands. However, renal dialysis for chronic renal failure commenced in 1960 after the successful development of HD [2]. Consequently, improving the HD technique to develop sufficient arteriovenous fistula, which occurred in 1972, allowed for faster expansion of dialysis services [3]. After 1972, HD equipment was designed to provide long-lasting ambulatory peritoneal dialysis and home-care hemodialysis. The improvement in renal replacement therapy encompassed the presence of recombinant human erythropoietin, effective antihypertensive drugs, and calcitriol. Furthermore, technical development in HD included using biocompatible membranes, ultrafiltration, and membranes of higher porosity [4].

Dialysis services were introduced in Saudi Arabia (SA) in the early 1970s [5]. Since then, dialysis centers have expanded across different regions of the country, with growth influenced by service capacity and geographical coverage. The overall economic development of SA has played a pivotal role in the nationwide expansion of these services. Many units have upgraded to advanced hemodialysis machines, allowing for the adoption of bicarbonate dialysis, controlled ultrafiltration, and sodium profile modeling. Additionally, in the last two decades, a more comprehensive range of biocompatible dialyzers has become available [6].

As reported by Shaheen et al., there has been a rise in the number of dialysis centers in Saudi Arabia. Among these, 55% are associated with Ministry of Health (MoH) hospitals. Government non-MoH hospitals contribute 9%, while 13% result from outsourced dialysis initiatives. Charitable and private hospitals comprise 21%, and the King Abdullah Hemodialysis Project accounts for 2%. In 2019, there were 243 dialysis centers in the country, but this number has since increased. The distribution of these centers is uneven, with the central region having the highest share at 30%, followed by the western region at 27%, the southern region at 19%, the eastern region at 11%, and the northern region at 11%. Furthermore, Shaheen et al. noted an annual increase of 6.2% in dialysis patients, projected to reach 22,000 by 2020 [7]. In 2021, the actual number surpassed 20,000, and 9,810 sick people were undergoing medication following kidney transplantation [4]. The demand for renal replacement treatment in Saudi Arabia was estimated to involve 294.3 million people in 2021 [8].

The Saudi government has dedicated resources to constructing and enhancing healthcare infrastructure, incorporating hospitals and medical facilities that feature cutting-edge technologies for diagnosis and treatment [9]. Notably, several government-launched initiatives contribute to the enhancement of healthcare services nationwide. These initiatives include Saudi Vision 2030, the National Transformation Program (NTP), compulsory health insurance, and the Saudi Center for Organ Transplantation (SCOT). These efforts play a significant role in advancing dialysis services in Saudi Arabia [10]. Therefore, examining how people utilize renal dialysis in MoH, other governmental sector hospitals, and private healthcare sectors is essential. For this reason, the following hypotheses were tested:

### H_0_

A positive relationship exists between the health sector (HS) and the number of renal dialysis centers and peritoneodialysis patients.

### H_1_

There is a negative relationship between health sectors (HS), renal dialysis centers, and peritoneodialysis patients.

## Methods

### Research design

1. This study aimed to comprehend patient outcomes and diverse practices of renal dialysis in various healthcare settings in Saudi Arabia using a quantitative research design. Descriptive research design was used as one of the quantitative research types, focusing on providing a detailed account of a phenomenon or population [11]. This design involves the collection and analysis of data without manipulating variables. The primary objective of descriptive research is to observe, record, and report on what is happening or existing without attempting to establish cause-and-effect relationships, which is suitable for the current study [12]. Furthermore, a quantitative observational study was conducted, wherein the research analyzed and extracted valuable information about renal dialysis practices by examining data from the General Authority for Statistics (GaStat) of Saudi Arabia and the MoH (Chapter IV: Health Activities and Services) indicators for Renal Dialysis Centers and Patients across Health Sectors in the year 2021 [13].

### Sampling strategy

The research objectives were achieved using secondary data from the General Authority of Saudi Arabia and the MoH (Chapter IV: Health Activities and Services) indicators for Renal Dialysis Centers and Patients by Health Sectors in 2021 [14]. The inclusion criteria considered variations in the healthcare sector (comprising the Ministry of Health, other government sectors, and the private sector) and patient demographics. Moreover, non-health-related data were excluded from the study. This approach incorporated a comprehensive and diverse dataset for subsequent analysis. Additionally, purposive sampling was employed, involving selecting data based on criteria that align with the research objectives.

### Data collection

Structured observation was employed to gather data from the General Authority for Statistics (GaStat) of Saudi Arabia and the MoH (Chapter IV: Health Activities and Services) indicators for Renal Dialysis Centers and Patients by Health Sectors in 2021 [15]. This data was then transferred to MS Excel 365 for subsequent analysis. The data underwent coding based on various factors, including health sectors, gender, nationality, hemodialysis by the health sector, renal dialysis by the health sector, number of dialysis centers, number of hemodialysis machines, number of hemodialysis patients, and number of peritoneodialysis patients.

### Variables and measures

The dependent variable in this study was the healthcare sector. Conversely, the independent variables included hemodialysis patients categorized by the health sector, gender, and nationality, and renal dialysis centers and patients classified by the health sector. Additionally, the measures employed encompassed age, gender, ethnicity, the number of renal dialysis centers, the number of hemodialysis machines, the number of hemodialysis patients, and the number of peritoneodialysis patients.

### Procedure

Information was acquired from the General Authority for Statistics (GaStat) of Saudi Arabia and the MoH (Chapter IV: Health Activities and Services) Indicators for Renal Dialysis Centers and Patients by Health Sectors in 2021. Subsequently, the data underwent coding and appropriate formatting within MS Excel 365. The organized data was then utilized to generate pertinent tables and graphs using MS Excel 365. However, the interrelationship between dependent and independent variables was analyzed through SPSS Version 29 (multiple regression analysis).

### Data analysis

Data analysis was conducted using MS Excel 365 and SPSS Version 29, using a confidence level of 95%, with a desired power of 80% and a two-tailed approach. The data were summarized based on the distribution of machines and patients across health sectors, renal dialysis centers within health sectors, trends related to gender, and correlations. Additionally, the analysis delved into aspects such as nationality, renal dialysis centers, and patients distributed across health sectors.

### Subjects

The study subjects encompassed hemodialysis patients categorized by nationality, gender, and health sectors, along with renal dialysis centers classified by healthcare sectors and peritoneodialysis patients.

### Reliability and validity

As secondary data was utilized, the reliability and validity of the data depended on the primary data source. The analysis relied on the reliability and validity approach employed in the original data collection. Data were derived from the official agency.

### Statistical analysis (interpretation)

Statistical analysis, including MS Excel 365 and SPSS Version 29, were utilized jointly as the leading software programs for data analysis. Specifically, multiple linear regression was used to determine the relationship between the dependent variable (health sector) and independent variables. This analytical process yielded responses to the research inquiries. However, the collinearity test was conducted to determine independent variables which are closely related.

## Results

Table 1 illustrates 275 renal dialysis centers, over 8000 HD machines, 20,440 HD patients, and 1,861 peritoneal patients. Ministry of Health facilities had the highest number of renal dialysis centers (70.18%), followed by the private sector (18.55%) and then another government sector (11.27%). Furthermore, MoH had the highest number of hemodialysis machines (67.13%) compared to other governmental industries (19.61%) and the private sector (13.26%). Non-Saudis received more of their renal dialysis services from the private sector (63.52%) than from another governmental industry (8.65%) and MoH (7.2%) (Table 1).

**Table 1:** Descriptive statistics for the associated variables of renal dialysis services

Provider/Statistics	RDC		HM		HP								PP	
					NS		S		Total		Overall			
	No.	(%)	No.	(%)	M	F	M	F	NS	S	No.	(%)	No.	(%)
MoH	193	70.2	5,437	67.13	946	125	11,205	99	1,071	11,304	12,375	60.54	806	43.3
Other Governmental Sector	31	11.3	1,588	19.607	375	99	3958	153	474	4111	4585	22.43	1,055	56.7
Private Sector	51	18.5	1,074	13.26	1947	63	1118	352	2010	1470	3480	17.03	0	0
Total	275	100	8,099	100	3268	287	16,281	604	3,555	16,885	20,440	100	1,861	100

RDC = Renal Dialysis Centers; HM = Hemodialysis Machines; HP = Hemodialysis Patients; PP = Peritoneodialysis Patients

**Fig. 1:**
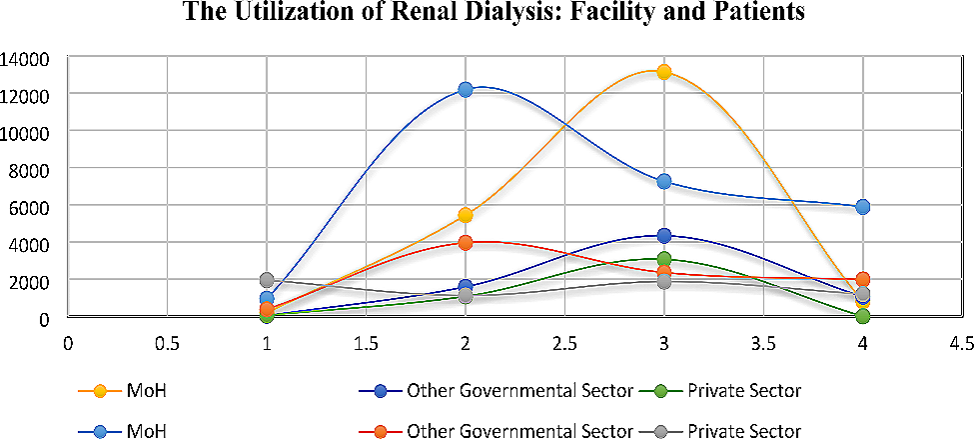
Facility allocation and patients attending several types of healthcare services

Contrary to non-Saudi Arabians, Saudi Arabians preferred MoH (64%) for the majority of their renal dialysis services over other governmental sectors (21%) and the private sector (14.93%) (Fig. 1).

Across the three healthcare sectors, the number of males (55.21% for MoH, 54.28% for other governmental sector, and 60.94% for private sector) seeking renal dialysis services was more than the number of females (44.79% for MoH, 45.72% for other governmental sector, and 39.053% for private sector) (Table 1, and Fig. 1). Surprisingly, peritoneodialysis patients only sought renal dialysis services from MoH and other governmental sectors—most visited other governmental sectors (56.69%) than MoH (43.3%).

Table 2, HS characterizes the health sector (three healthcare sectors), NRDC defines the number of renal dialysis centers, NHM denotes the number of hemodialysis machines, NS signifies Non-Saudi Arabians, S represents Saudi Arabians, and M is the number of males. At the same time, F describes the number of females. Moreover, NPP is the number of peritoneodialysis. NRDC and NPP have relatively high Tolerance values (0.967), indicating that around 96.7% of their variance is not explained by the other predictor variables in the model. The VIF values for NRDC and NPP are close to 1 (1.034), suggesting minimal multicollinearity between these variables. VIF values close to 1 indicate that the variables are not highly correlated.

On the other hand, NHM, NS, S, M, and F have Tolerance values of 0, indicating that all of their variance is explained by the other predictor variables in the model. This shows a high degree of multicollinearity. Furthermore, the VIF values for NHM, NS, S, M, and F are also 0, which is expected when the Tolerance values are 0. VIF values of 0 indicate perfect multicollinearity, meaning these variables are perfectly linearly dependent on the other predictors in the model. For this reason, only NRDC and NPP were the independent variables used in the analysis, leading to Eq. 1.

1 $$\mathrm{HS}=\beta 0+\beta 1 \mathrm{NRDC}+\beta 2 \mathrm{NPP}+\varepsilon$$

**Table 2:** Collinearity test between independent variables (NPP, NRDC, NHM, NS, S, M, and F)

	Tolerance	VIF
NRDC	0.967	1.034
NPP	0.967	1.034
NHM	0	0
NS	0	0
S	0	0
M	0	0
F	0	0

Based on the data in Table 3, Eq. 1 becomes as demonstrated in Eq. 2. Equation 2.

Equation 2 provides insight into how the number of renal dialysis centers (NRDC) and the number of peritoneodialysis patients (NPP) in the health sector (HS). The intercept term 3.401 represents the expected HS when NRDC and NPP are zero. The negative coefficient of − 0.694 for NRDC suggests that for each number of renal dialysis centers, there is an anticipated decrease of 0.694 units in the health sector, holding the number of peritoneodialysis patients constant. Similarly, the negative coefficient of − 0.605 for NPP indicates that with each one-unit increase in the percentage of peritoneodialysis patients, there is an expected decrease of 0.605 units in the health sector, holding the number of renal dialysis centers constant. Therefore, according to this regression analysis, an increase in renal dialysis centers and peritoneodialysis patients is associated with a decrease in the health sector. The findings reveal that the number of renal dialysis centers and peritoneodialysis are not equally distributed in the health sector, and the relationship is statistically significant (p-value < 0.05), leading to the rejection of the null hypothesis.

2 $$\mathrm{HS}=3.401-0.694 \mathrm{NRDC}-0.605 \mathrm{NPP}$$

**Table 3:** The relationship between HS, NRDC, and NPP

				95% CI for B		
Model		B	*P*-value	Lower Bound	Upper Bound	Std. Error
1	Constant	3.401		3.401	3.401	0
	NRDC	-0.694	<0.05	-0.008	-0.008	0
	NPP	-0.605	<0.05	-0.001	-0.001	0

## Discussion

The study examined the utilization of renal dialysis in 2021 in different healthcare sectors, including MoH, another governmental sector, and the private sector. As a result, the present research focused on the number of renal dialysis centers, the number of HD machines, the number of HD patients, measured according to nationality and sex, and the number of peritoneodialysis patients to determine how people utilize renal dialysis in MoH, other governmental sectors, and private sector. Descriptive and inferential statistics were conducted to define the relationship between these variables. Descriptive statistics revealed the characteristics of the samples. On the other hand, inferential statistics (correlation) tested the hypotheses to assess if a relationship exists between the health sector (HS) and the number of peritoneodialyis patients.

### Utilization of renal dialysis services based on percentage

In 2021, MoH (70.18%) had the highest number of renal dialysis centers compared to the other governmental sector (11.27%) and the private sector (18.55%). Additionally, more HD machines were available at MoH (67.13%) than in other governmental industries (19.61%) and private sectors (13.26%). Based on these findings, more patients were seeking renal dialysis services from MoH (64%) than from other governmental industries (21%) and private sector hospitals (14.93%), demonstrating the availability of HD machines and renal dialysis centers influenced patients’ preference on specific healthcare sectors.

The results showed that a majority of Saudi Arabians (92.80%) preferred the Ministry of Health (MoH) for renal dialysis services compared to other governmental (91.34%) and private sector (36.48%) hospitals. There are several reasons underlying this preference for MoH over alternative healthcare providers. One key factor is that MoH facilities are government-funded, allowing them to offer renal dialysis services at a more affordable cost or even free charge. In contrast, private sector hospitals may impose higher fees for their services, making MoH hospitals a more cost-effective choice for individuals. Additionally, according to [14, 16, and 17], many residents in Saudi Arabia may possess health insurance plans that cover services offered by MoH hospitals. When private sector hospitals lack coverage or have limited benefits, individuals may choose MoH hospitals to minimize out-of-pocket expenses. Trust also plays a pivotal role in the decision-making process for Saudi Arabian citizens seeking renal dialysis services. This finding concurred with [18, 19] outcomes, which indicated that some individuals may place greater trust in the quality and reliability of healthcare services provided by government-related institutions. MoH hospitals are often perceived as having standardized procedures and protocols, fostering a sense of security among patients. Public perception is another factor contributing to the significant volume of patients utilizing dialysis services in Saudi Arabia.

Furthermore, many research studies argued that public hospitals are occasionally viewed as having healthcare professionals with more excellent expertise and better-equipped facilities, which supported the present findings [20–22]. This perception might influence individuals to opt for MoH or other governmental sector hospitals for renal dialysis services instead of private-sector hospitals. Alhamad et al. found that patients consider transportation means, family, and social support when selecting dialysis centers, aligning with the current findings consistent with the current outcome [23–25]. In line with Friberg et al.’s research, patients were more inclined towards home dialysis, receiving comprehensive and high-quality information about predialysis from three or more sources [26–28].

The findings established that more non-Saudi Arabia (63.52%) sought renal dialysis services from private sector hospitals than other governmental sector hospitals (8.65%) and MoH (7.2%). Factors such as insurance policies and preference coverage might have contributed to non-Saudis’ preference for private sector hospitals over other governmental sector hospitals and MoH. Moreover, the number of hemodialysis patients (91.69%) surpassed that of peritoneodialysis patients (8.31%). This discrepancy can be attributed to the broader availability and accessibility of hemodialysis as a form of renal replacement therapy across various healthcare settings. The infrastructure necessary for hemodialysis, including dialysis units and adequately trained staff, may also be more prevalent than peritoneal dialysis. Additionally, not all patients are viable candidates for peritoneal dialysis, with factors such as overall health, abdominal condition, and willingness to engage in self-care at home influencing the preference between hemodialysis and peritoneal dialysis [29–32]. Certain patients may have medical conditions or lifestyle considerations that make hemodialysis more suitable.

Due to resource and infrastructure limitations, peritoneodialysis patients were notably absent in private-sector hospitals. This limitation arises from the specific requirements for establishing and sustaining a peritoneal dialysis program, including the need for trained staff, specialized equipment, and infrastructure to support home-based care [6, 33, 34]. Private sector hospitals may allocate resources based on services with higher demand or those more commonly sought by their patient population. Additionally, insurance coverage considerations may influence the absence of peritoneodialysis in private-sector hospitals [8, 9, 35, 36]. In Saudi Arabia, insurance coverage tends to support hemodialysis more than peritoneal dialysis, prompting patients to choose the financially more viable option [41 and 42].

### The relationship between HS, and NRDC, and NPP

The study tested the relationship between the number of renal dialysis centers and the number of peritoneodialysis patients. According to the present findings, *p* < 0.05 leads to rejecting the null hypothesis (H_0_), indicating a negative relationship between the number of renal dialysis centers and peritoneodialysis patients. Therefore, the number of renal dialysis centers and peritoneodialysis differs in the health sector (MoH, another government sector, sectors sector). The uneven distribution of renal dialysis centers and peritoneodialysis patients is due to machine availability and people’s preferences. The findings supported [37–39, and 40], which found the same results.

### Limitations

This study relied on secondary data sources, subject to several limitations. For instance, certain facets of the research question have yet to be covered during the initial data collection, resulting in information gaps. Moreover, the data may be aggregated at a level that aligns differently from the specific details required for the present study. Additionally, the primary data might not have included procedures for obtaining informed consent, necessitating the researcher to consider ethical implications carefully.

## Conclusions

The study assessed the utilization of renal dialysis services across different healthcare sectors in Saudi Arabia, focusing on the number of renal dialysis centers, HD machines, and HD and peritoneodialysis patients. The prevailing healthcare policies support Saudi Arabian citizens for renal dialysis services within the Ministry of Health (MoH) sector and its affiliated hospitals over private sector facilities. This preference is supported by a higher attendance of hemodialysis patients in the MoH sector and its associated hospitals. Moreover, substantial investments in MoH sector hospitals in Saudi Arabia have resulted in more dialysis machines and superior, more affordable healthcare services than private-sector hospitals. Notably, discernible variations in the quality of care and patient outcomes exist across these three healthcare sectors, as demonstrated by multiple regression analysis.

The government funding assists MoH sector hospitals and affiliated hospitals in offering high-quality renal dialysis at a reasonable cost. In contrast, private sector hospitals provide quality renal dialysis services at a higher expense. The current healthcare policies in Saudi Arabia should also support non-Saudi residents in accessing renal dialysis services from the MoH sector and its affiliated hospitals. The key factors contributing to the disparities in effectiveness and efficiency among these healthcare sectors are Saudi Arabia’s healthcare policies, including insurance coverage policies, which are disadvantageous for non-Saudi residents.

## Data Availability

The dataset and the accompanying files are available from the General Authority for Statistics (GaStat) of Saudi Arabia (https://www.stats.gov.sa/en), and the Ministry of Health (MoH) (Chapter IV: Health Activities and Services) indicators for Renal Dialysis Centers and Patients by Health Sectors in 2021 )https://www.moh.gov.sa/en/Ministry/Statistics/book/Pages/default.aspx(
